# Adrenocortical Carcinoma With Cushing’s Syndrome and Hyperandrogenism in a 28-Year-Old Pregnant Female

**DOI:** 10.1016/j.aace.2023.03.002

**Published:** 2023-03-17

**Authors:** Michael John Marino, Sara Markley Webster

**Affiliations:** 1 Department of Graduate Medical Education, Northside Hospital Gwinnett, Lawrenceville, Georgia; 2 Division of Endocrinology, Department of Internal Medicine, Emory University School of Medicine, Atlanta, Georgia; 3 Atlanta Veterans Affairs Medical Center, Atlanta, Georgia

**Keywords:** adrenocortical carcinoma, Cushing’s syndrome, hyperandrogenism, pregnancy, mitotane

## Abstract

**Background/Objective:**

To describe a case highlighting a rare malignancy that can be camouflaged by the hormonal milieu of pregnancy.

**Case Report:**

We present the case of a 28-year-old pregnant female who was diagnosed with stage IV metastatic adrenocortical carcinoma at 15-weeks gestation. The patient declined palliative chemotherapy at first with the hope of continuing her pregnancy. She had elevated dehydroepiandrosterone sulfate, testosterone, and cortisol levels consistent with Cushing’s syndrome and hyperandrogenism. The patient eventually had a spontaneous abortion and elected to start chemotherapy and mitotane treatment. She passed away 3 months after initial presentation.

**Discussion:**

Adrenocortical carcinoma is difficult to detect and diagnose in pregnant patients because of the physiologic hormonal changes that take place during gestation. The patient described in this case report is an example of this diagnostic challenge.

**Conclusion:**

Adrenocortical carcinoma is a rare, fatal disease that often presents at an advanced stage with limited treatment options making earlier diagnosis imperative; however, diagnosis and treatment are complicated by pregnancy. More data is necessary to determine how best to approach these challenges in future patients.

**Highlights**

• Adrenocortical carcinoma is a very rare cancer with a poor prognosis.
• Diagnosing adrenocortical carcinoma during pregnancy is difficult due to hormonal changes of pregnancy.
• Surgical resection is the gold standard.
• Cytotoxic chemotherapy agents such as mitotane have modest efficacy.

**Clinical Relevance Summary**

All cases of adrenocortical carcinoma are clinically relevant and should be reported due to the rarity of this cancer. This case report will add to the existing literature and help to further the understanding of the diagnosis, treatment, and clinical course of adrenocortical carcinomas.

## Introduction

Adrenocortical carcinoma (ACC) is an exceptionally rare disease with a poor prognosis. ACC has an incidence of 0.72 per million cases in the United States and affects females at a 2:1 ratio.^1^^,^^2^ Diagnosing ACC during pregnancy becomes even more difficult because the symptoms associated with ACC mimic those of normal pregnancy or its complications.^3^ Unfortunately, a majority of patients are found to have metastatic disease at the time of their initial diagnosis. Surgical resection is the preferred treatment even in patients with metastatic disease and cytotoxic chemotherapy agents such as mitotane are used with modest efficacy reported.^4^

## Case Report

A 28-year-old G7P0060 female at 15 weeks, 6 days gestation was admitted for hypertensive crisis. At 7-weeks gestation, she had normal vitals. At 12 weeks, proteinuria and hypertension were noted. After dietary modifications, the next visit revealed resolved proteinuria but worsened hypertension, for which she was admitted. The patient reported intermittent sharp left flank pains over the last 2 years, which worsened during pregnancy. She noted dyspnea, palpitations, and facial hair growth during the pregnancy. She reported no diaphoresis, unexplained weight changes, nor striae/acne. Initial workup revealed labs as noted in Table 1.

**Table 1:** Admission Laboratory Values

Lab ordered	Lab value	Reference range
Free normetanephrine	0.34 nmol/L	<0.9 nmol/L
Free metanephrine	0.10 nmol/L	<0.5 nmol/L
Cortisol	116.6 mcg/dL	0-25 mcg/dL, (7-19, 10-42 mcg/dL during first and second trimester, respectively)
Sodium	132 mmol/L	135-145 mmol/L
Potassium	3.4 mmol/L	3.5-5.1 mmol/L
Bicarbonate	21 mmol/L	24-31 mmol/L
Calcium	8.8 mg/dL	8.5-10.3 mg/dL
Lactate dehydrogenase	880 IU/L	140-280 IU/L
Alkaline Phosphatase	163 IU/L	40-150 IU/L

Renal ultrasound revealed a left suprarenal mass and magnetic resonance imaging detailed a 12 × 10 × 8cm adrenal mass with innumerable lung nodules. Biopsies were requested by oncology and general surgery to determine what treatment options they might offer. Biopsy of the suprarenal mass showed extensive necrosis with fragments of adrenal cortical tissue consistent with adrenocortical neoplasm. Immunohistochemical staining demonstrated a high Ki-67/MIB proliferative index, focal inhibin staining, and diffuse synaptophysin positivity. Lung nodule biopsy showed malignant cells with inhibin/synaptophysin positivity and CD68 negativity, consistent with metastatic ACC. Given the severe metastatic lung burden, adrenalectomy was not offered during hospitalization.

As seen in Table 2, the patient lacked diurnal variation in serum cortisol levels, which is usually preserved in pregnancy. These labs were drawn while the patient was hospitalized, which potentially affected the results secondary to high-stress environment; mitigation was attempted by repeating testing on multiple days. Of note, cortisol levels are known to follow a gradual increase throughout pregnancy, peak at delivery, and have a sharp decline to baseline within the first 3 days postpartum.^5^ Dehydroepiandrosterone sulfate and testosterone levels were severely elevated, consistent with adrenal hyperandrogenism (Table 3). 17-hydroxyprogesterone was elevated beyond expected for the patient’s stage of pregnancy, where levels up to 941 ng/dL can be seen in the first and second trimesters and levels up to 2772 ng/dL in third trimester.

**Table 2:** AM/PM Serum Cortisol Levels

Hospital Day	Lab	Value	Reference Range
2	Midnight serum cortisol	64.5 mcg/dL	
3	AM serum cortisol	62.2 mg/dL	5-25 mcg/dL
3	Midnight serum cortisol	48.9 mcg/dL	
5	Midnight serum cortisol	61.5 mcg/dL	
5	Midnight salivary cortisol	3.875 ug/dL	(ref: for 19-30 year old patients, <0.359 ug/dL)
6	AM serum cortisol	53.9 mcg/dL	5-25 mcg/dL
12	24 h free urine cortisol	1240.6 mcg/24 h	<45 mcg/24 h
12	AM serum cortisol	61.6 mcg/dL	5-25 mcg/dL
25 (PPD 7)	AM serum cortisol	59.0 mcg/dL	5-25 mcg/dL
32 (outpatient)	AM serum cortisol	63.9 mcg/dL	5-25 mcg/dL
39 (readmission d)	AM serum cortisol	63.7 mcg/dL	5-25 mcg/dL
42 (1 d after mitotane initiation)	AM serum cortisol	44.1 mcg/dL	5-25 mcg/dL

**Table 3:** Laboratory Profile Suggestive of Hyperandrogenism

Lab	Initial Value	Postpartum Day 1/Hospital Day 19 Value	Reference Range
Dehydroepiandrosterone sulfate	>1000 mcg/dL	>1000 mcg/dL	35-430 mcg/dL
Total testosterone	224 ng/dL	73 ng/dL	2-34 ng/dL
Free testosterone	36.8 pg/mL	23.0 pg/mL	2.5-6.4 pg/mL
17-hydroxyprogesterone	1140 ng/dL	942.00 ng/dL	<206 ng/dL

The patient was diagnosed with Stage IV adrenocortical carcinoma with Cushing’s syndrome (CS) and hyperandrogenism. Palliative chemotherapy after abortion was offered but treatment was deferred in hopes of viable pregnancy. A spontaneous abortion occurred at 17-week gestation.

Postpartum day 1/hospital day (HD) 19 labs are noted in Table 3. HD 25 serum cortisol was 59 mcg/dL while on dexamethasone 2 mg twice a day for back pain secondary to bone metastases. Given the continued unsuppressed serum cortisol and ACTH levels on dexamethasone 2 mg twice a day, CS was confirmed, despite absent Cushingoid appearance.

After fetal demise, palliative chemotherapy was planned. The regimen included cisplatin, etoposide, adriamycin, mitotane, and metyrapone for CS. The patient declined treatment initiation inpatient and was discharged on HD 26.

She presented on HD 39 with dyspnea and edema and was readmitted. She began leuprolide for fertility preservation prior to chemotherapy. The patient was then started on mitotane 500 mg three times a day (increased to 1000 mg twice a day the following day, 1500 mg twice a day 1 week later, and 2000 mg twice a day 2 weeks later), metyrapone 250 mg three times a day (1 week later increased to Q6h), and spironolactone 50 mg daily for androgen excess. The patient’s labs after initiation of mitotane and metyrapone are noted in Table 4. Mitotane ultimately was discontinued due to GI symptoms without reaching therapeutic range.

**Table 4:** Labs 2 Weeks After Initiation of Mitotane and Metyrapone

Lab	Initial value	Reference range
Adrenocorticotropic hormone	15 pg/mL	10-60 pg/mL
AM serum cortisol	24.9 mcg/dL	5-25 mcg/dL
24 h urine cortisol	37.2 mcg/24 h	<45 mcg/24 h
Mitotane	1.9 mg/dL	14-20 mg/dL (therapeutic range)

A repeat computed tomography revealed an enlarging necrotic/hemorrhagic left adrenal mass 10.6 × 14.3 × 16.3cm, with renal and diaphragmatic invasion. Chemotherapy started on HD 44 with cisplatin, etoposide, and adriamycin. While on chemotherapy, the patient was started on high dose dexamethasone (12 mg daily) due to concerns for adrenal insufficiency while on mitotane.

The patient was on day 1 of chemotherapy cycle two when the chemotherapy was stopped for hospice initiation. She died 3 months after initial presentation, after undergoing 31 days of mitotane and 1 cycle of chemotherapy. Genetic testing showed that the patient was heterozygous for the p.Q301H variant of unknown significance in the protection of telomeres 1 gene. Variants in this gene have been associated with a predisposition to certain types of cancers.

## Discussion

Adrenocortical carcinomas represent a small percentage of adrenal tumors and have a low worldwide incidence.^6^ The 5-year survival rate is around 30%, representing a grim prognosis.^7^ The presentation of ACC most commonly involves CS with virilization.^8^ CS during pregnancy causes maternal hypertension in 68% of cases, maternal death in 2% of cases, and spontaneous abortion/intrauterine death in 5% of cases.^9^ It is reported that benign and malignant adrenocortical tumors represent 70% of all cases of CS diagnosed during pregnancy.^10^

Diagnosing CS of any cause during pregnancy is difficult because pregnancy produces changes in the hypothalamic-pituitary-adrenal axis. The pregnant state causes increased production of cortisol-binding globulin and elevated levels of cortisol. Additionally, the placenta produces increased levels of adrenocorticotropic hormone and corticotropin releasing hormone. One study revealed urinary free cortisol in each trimester of pregnancy were 135 nmol/d, 187 nmol/d, and 240 d nmol/d compared to 78 nmol/d in nonpregnant patients.^11^ Cortisol suppressibility by dexamethasone is less effective in pregnancy and is increasingly less effective as pregnancy advances.^12^ Diurnal variation of cortisol during pregnancy is preserved in plasma and salivary cortisol levels.^13^ Absent diurnal variation remains useful in establishing CS in pregnancy, particularly as few studies have evaluated late night salivary cortisol (LNSC) levels in pregnancy. Lopes et al attempted to establish reference ranges for LNSC levels by comparing LNSC levels in pregnant patients, nonpregnant patients, and nonpregnant patients with CS, finding a 1.1-, 1.4-, and 2.1-fold increase in LNSC by trimester.^14^ A reasonable option for diagnosis would be to establish highly elevated LNSC levels and lack of diurnal cortisol variation; however, there are currently no definitive guidelines in the literature to guide diagnosis of CS during pregnancy. If CS is diagnosed during pregnancy treatment options include surgical resection or medical management with metyrapone, an enzyme inhibitor that blocks the formation of cortisol.^15^

Given the rare nature of ACC, very little is known about ACC in pregnant patients. According to a 2010 publication by Abiven-Lepage et al in the European Journal of Endocrinology, there were <20 cases of ACC during pregnancy described to date.^16^ This is corroborated by the 2014 case report and literature review published by Jarvis and Morton stating that there are <180 cases of CS reported in pregnancy and <20 cases of ACC reported.^17^ This is partly due to women with ACC having lower fertility at baseline due to the effects of hypercortisolism and/or hyperandrogenism.^16^ In a retrospective cohort study by Abiven-Lepage et al, the authors reviewed 110 female patients aged 16- to 49-year-old who were diagnosed with ACC. Twelve of these patients were either pregnant or within 6 months postpartum. The pregnant/postpartum subset of patients had younger mean age at diagnosis, larger tumor size, and more advanced disease than nonpregnant patients. Two thirds of the pregnant/postpartum subset of patients had local or metastatic extension compared to two thirds of the nonpregnant group with tumor limited to the adrenal gland.^16^ Also, 100% of the tumors diagnosed in the pregnancy/postpartum group secreted cortisol compared to 74% in the nonpregnant group.^16^

Interestingly, none of the women died during pregnancy in the Abiven-Lepage et al study; however, the overall survival rate remained low for these ACC patients when compared to a subgroup of nonpregnant women matched for age, stage, and year of diagnosis. There was 13% survival at the 5 year mark and 0% survival at 8 years in pregnant ACC patients, compared with 50% survival in nonpregnant ACC at both time points.^16^ Maternal complications include hypertension, diabetes, preeclampsia, and more.^18^ Fetal outcomes include premature births, stillbirths, intrauterine growth retardation, adrenal hypoplasia, and spontaneous abortion.^16^^,^^18^

If surgery is indicated, the best time for intervention is the second trimester.^19^ Mitotane, an adrenolytic drug that causes destruction of the inner zones of the adrenal cortex, is the only drug approved by the FDA.^20^ Mitotane affects the adrenal cortical cell mitochondria thereby inhibiting pregnenolone and cortisol synthesis as well as altering the peripheral metabolism of steroids.^21^ Mitotane is often used in patients with inoperable tumors as well as adjuvant therapy after surgical resection. GI side effects are common.^2^ Mitotane has been found to prolong survival slightly but not significantly.^4^ Notably, mitotane is not recommended during pregnancy and concomitant contraception is recommended.^21^ In women who previously had ACC and received treatment, subsequent pregnancies do not increase the risk of recurrence or death.^10^ It is recommended to wait until the mitotane plasma level reaches undetectable levels (1-3 years) before pursuing pregnancy.

In summary, clinicians should remember to consider CS in pregnant patients who present with secondary hypertension, particularly if there are other clinical symptoms or lab abnormalities consistent with this diagnosis, such as hirsutism or hypokalemia, both of which were seen in this patient. Checking serum cortisol levels and noting a lack of circadian rhythm in cortisol levels can be an important clue that points toward the diagnosis of CS. Although rare, clinicians should remember to evaluate for ACC in the setting of CS, particularly when hyperandrogenism is coexisting. Although patients with ACC have a poor prognosis, faster diagnosis could increase chances of surgical resection and improve overall outcomes from this rare cancer.

## Disclosure

The authors have no multiplicity of interest to disclose.
